# Efficacy and safety of ketone ester infusion to prevent muscle weakness in a mouse model of sepsis-induced critical illness

**DOI:** 10.1038/s41598-022-14961-w

**Published:** 2022-06-22

**Authors:** Ruben Weckx, Chloë Goossens, Sarah Derde, Lies Pauwels, Sarah Vander Perre, Greet Van den Berghe, Lies Langouche

**Affiliations:** Clinical Division and Laboratory of Intensive Care Medicine, Department of Cellular and Molecular Medicine, KU Leuven, Herestraat 49, O&N1 bus 503, 3000 Leuven, Belgium

**Keywords:** Toxicology, Metabolic disorders

## Abstract

In septic mice, 3-hydroxybutyrate-sodium-salt has shown to partially prevent sepsis-induced muscle weakness. Although effective, the excessive sodium load was toxic. We here investigated whether ketone ester 3-hydroxybutyl-3-hydroxybutanoate (3HHB) was a safer alternative. In a mouse model of abdominal sepsis, the effects of increasing bolus doses of 3HHB enantiomers on mortality, morbidity and muscle force were investigated (n = 376). Next, plasma 3HB^-^ clearance after bolus d-3HHB was investigated (n = 27). Subsequently, in septic mice, the effect on mortality and muscle force of a continuous d,l-3HHB infusion was investigated (n = 72). In septic mice, as compared with placebo, muscle force was increased at 20 mmol/kg/day l-3HHB and at 40 mmol/kg/day d- and d,l-3HHB. However, severity of illness and mortality was increased by doubling the effective bolus doses. Bolus 3HHB caused a higher 3HB^−^ plasma peak and slower clearance with sepsis. Unlike bolus injections, continuous infusion of d,l-3HHB did not increase severity of illness or mortality, while remaining effective in improving muscle force. Treatment of septic mice with the ketone ester 3HHB partly prevented muscle weakness. Toxicity of 3HHB administered as bolus was completely avoided by continuous infusion of the same dose. Whether continuous infusion of ketone esters represents a promising intervention to also prevent ICU-acquired weakness in human patients should be investigated.

## Introduction

The majority of prolonged critically ill patients develop muscle weakness and wasting during their stay at the intensive care unit (ICU), which impairs recovery and is associated with longer-term morbidity and mortality^1–3^. Providing parenteral nutrition (PN) to supplement insufficient enteral nutrition was found unable to prevent this debilitating condition, whereas accepting a macronutrient deficit early during critical illness has shown to accelerate recovery and reduce muscle weakness^4,5^. In pediatric ICU patients, accepting such an early macronutrient deficit increased plasma concentrations of the ketone body 3-hydroxybutyrate, a direct effect that statistically mediated an important part of the outcome benefits^6,7^. In a mouse model of sepsis-induced prolonged critical illness, treatment with 3-hydroxybutyrate in the context of providing PN has previously shown to attenuate muscle weakness^8^. Remarkably, the muscle protection of 3-hydroxybutyrate was limited to a direct effect on muscle force, whereas the ongoing sepsis-induced muscle atrophy was not affected^8^. The ketone body protection did not appear to be related to its use as an energy substrate, but rather by its effects as a signaling molecule affecting regeneration pathways and by its conversion to cholesterol^8,9^.

**Figure 1:**
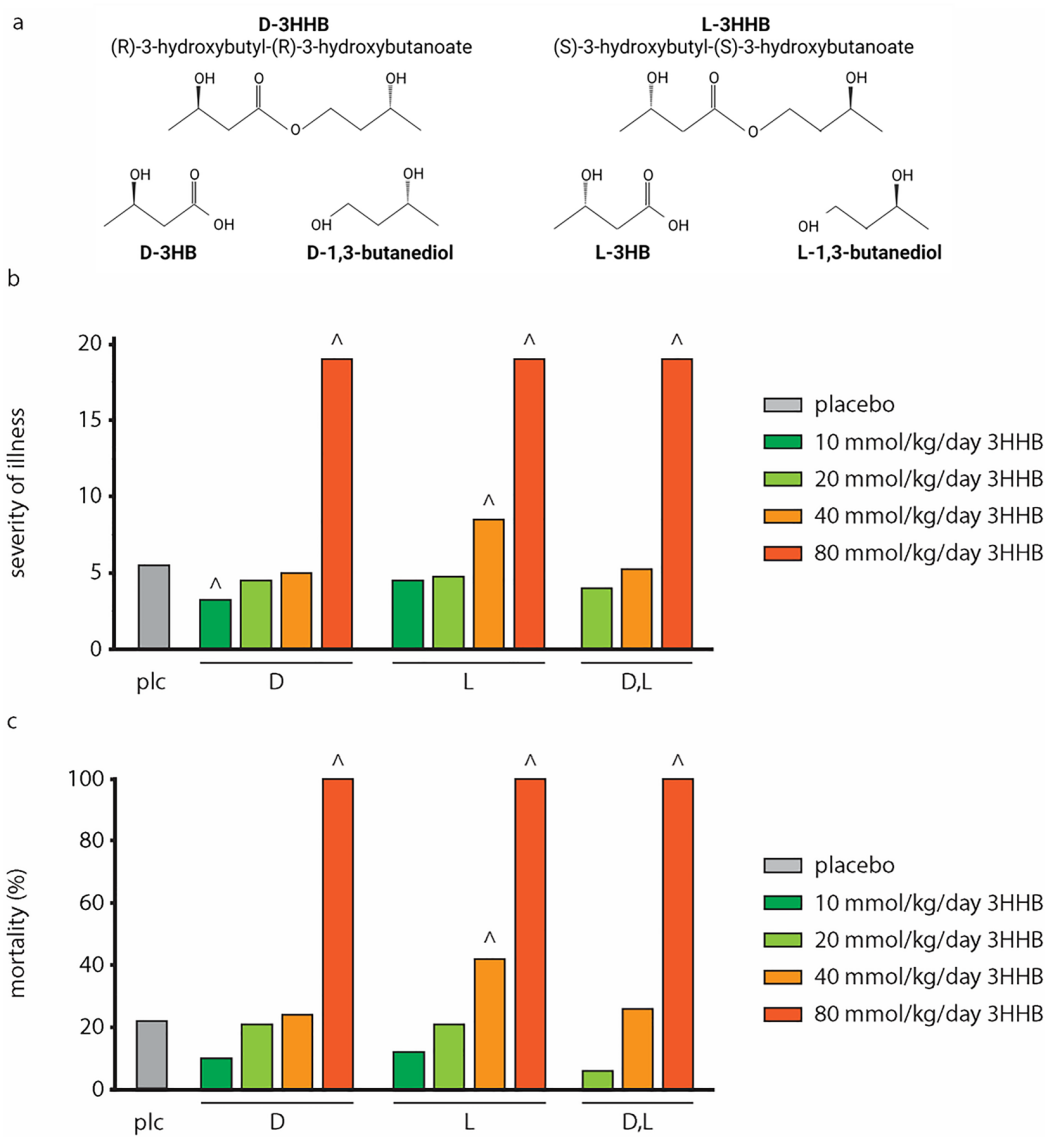
Impact of increasing bolus doses of pure and mixed racemic enantiomers of 3HHB on severity of illness and mortality. (**a**) Structure of 3HHB ketone esters and metabolites 3HB and 1,3-butanediol. (**b**) Cumulative severity of illness. Data are shown as medians. Number of animals equals total number of animals reported in (**c**). (**c**) Cumulative study mortality. Number of animals is reported as n = non-survivors/total. healthy control (n = 0/80); placebo (n = 23/103); d-3HHB: 10 mmol/kg/day (n = 2/20); 20 mmol/kg/day (n = 5/24); 40 mmol/kg/day (n = 5/21); 80 mmol/kg/day (n = 8/8); l-3HHB: 10 mmol/kg/day (n = 2/17); 20 mmol/kg/day (n = 5/24); 40 mmol/kg/day (n = 10/24); 80 mmol/kg/day (n = 8/8); d,l-3HHB: 20 mmol/kg/day (n = 1/17); 40 mmol/kg/day (n = 6/23); 80 mmol/kg/day (n = 7/7). *plc* placebo, *3HHB* 3-hydroxybutyl-3-hydroxybutanoate ester. ^^^p ≤ 0.05 vs placebo.

In these previous studies, the ketone body was administered as daily subcutaneous bolus injections of a racemic mixture of d,l-3-hydroxybutyrate sodium salt (3HB-Na). However, an escalating dose-finding study revealed that the effective dose of 40 mmol/kg/day of 3HB-Na was close to the toxic threshold^10^. Indeed, augmenting the daily dose with 20% or more increased severity of illness, markers of vital organ damage and mortality, in part explained by excessive Na^+^ intake. These adversities clearly preclude use of ketone salts in human critically ill patients. A potentially safer alternative for ketone salts is the ketone ester 3-hydroxybutyl-3-hydroxybutanoate (3HHB, Fig. 1a) which is endogenously converted to two 3HB^−^ molecules^11^. Sustained nutritional ketosis through oral ingestion of 3HHB has previously shown to be safe in healthy adults^12^ and has been extensively studied in elite athletes for its potential to improve exercise performance and endurance^13–15^.

Of note, 3HHB use in humans has been limited to the d-enantiomer of the ester, which is converted to d-3HB, the predominant endogenous enantiomer. However, also l-3HB is endogenously produced, albeit to a lesser extent and with a different utilization profile^16–18^. Tissue and plasma levels of l-3HB range between 5 and 23% of the total 3-HB concentration^19,20^. l-3HB displays a slower uptake and metabolization and is a favored substrate for the synthesis of sterols and fatty acids compared to d-3HB, which is preferentially oxidized^16,19^. These tissue specific differences in uptake and metabolization indicate that both enantiomers might have other beneficial effects^17,19^. In our previous experiments performed in septic mice, a racemic mixture of the 3HB salt was used and found effective to prevent muscle weakness^8,10^. A racemic 3HB mixture has also been investigated in humans suffering from congenital metabolic disorders and with the aim to improve cognition^21,22^. Studies comparing enantiomers of the ketone esters are however lacking.

The aim of the current study was to investigate whether the ketone ester 3HHB, administered either as d-3HHB, l-3HHB or the combination, was able to improve muscle force in parenterally fed, sepsis-induced critically ill mice, but with a better safety profile than the ketone salt 3HB-Na. To control for poor enteral absorption caused by the gastrointestinal dysfunction occurring during critical illness, ketone esters were administered by the parenteral route^23^. To determine the optimal method of administration in the context of critical illness, subcutaneous bolus injections and intravenous continuous infusion of 3HHB were tested.

## Results

### Effect of increasing bolus doses of pure and mixed racemic enantiomers of 3HHB on severity of illness scores and mortality

**Figure 2:**
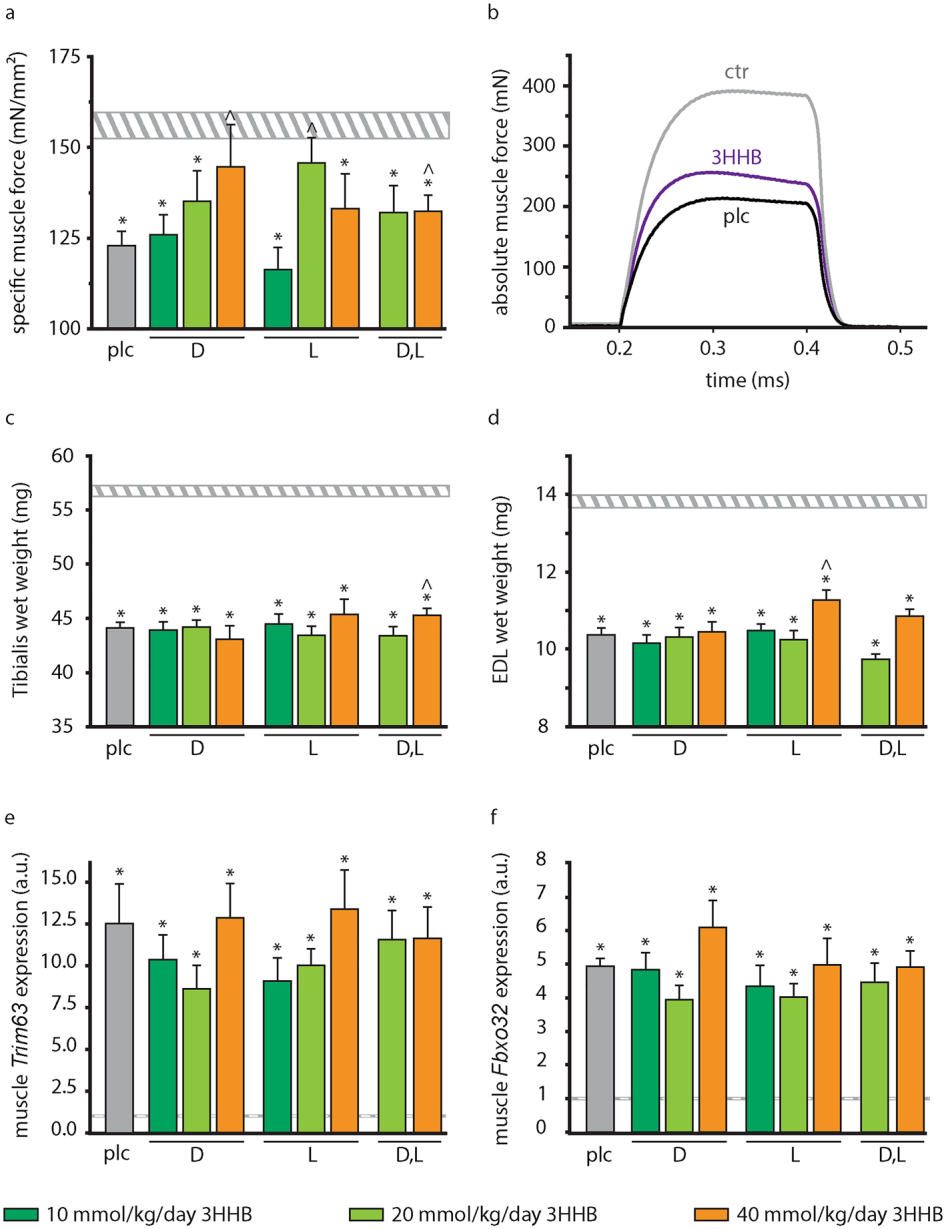
Impact of increasing bolus doses of pure and mixed racemic enantiomers of 3HHB on muscle force, muscle mass and muscle atrophy markers. (**a**) Ex vivo EDL force measurements presented as peak tetanic tension per unit muscle mass. Horizontal bars: white–grey shaded, healthy control (n = 62); grey, placebo (n = 67); vertical bars: d-3HHB: 10 mmol/kg/day (n = 17); 20 mmol/kg/day (n = 19); 40 mmol/kg/day (n = 16); l-3HHB: 10 mmol/kg/day (n = 15); 20 mmol/kg/day (n = 18); 40 mmol/kg/day (n = 14); d,l-3HHB: 20 mmol/kg/day (n = 15); 40 mmol/kg/day (n = 17). (**b**) Example of ex vivo-measured absolute maximal tetanic EDL muscle force presented as force tracings. 3HHB: 40 mmol/kg/day d,l-3HHB (**c**) Tibialis anterior and (**d**) EDL muscle wet weight. (**e**,**f**) Relative mRNA expression of muscle atrophy markers *Trim63* (**e**) and *Fbxo32* (**f**). Gene expression data are normalized to *Rn18s* and were expressed as a fold change of the mean of control mice. (**a**,**c-f**) Data are shown as mean ± standard error of the mean. Horizontal bar: healthy control (n = 80); vertical bars: placebo (n = 74); d-3HHB: 10 mmol/kg/day (n = 18); 20 mmol/kg/day (n = 19); 40 mmol/kg/day (n = 16); l-3HHB: 10 mmol/kg/day (n = 15); 20 mmol/kg/day (n = 19); 40 mmol/kg/day (n = 14); d,l-3HHB: 20 mmol/kg/day (n = 16); 40 mmol/kg/day (n = 17). *EDL* Extensor digitorum longus, *ctr* healthy control, *plc* placebo, *3HHB* 3-hydroxybutyl-3-hydroxybutanoate ester. *p ≤ 0.05 vs healthy control; ^^^p ≤ 0.05 vs placebo.

**Figure 3:**
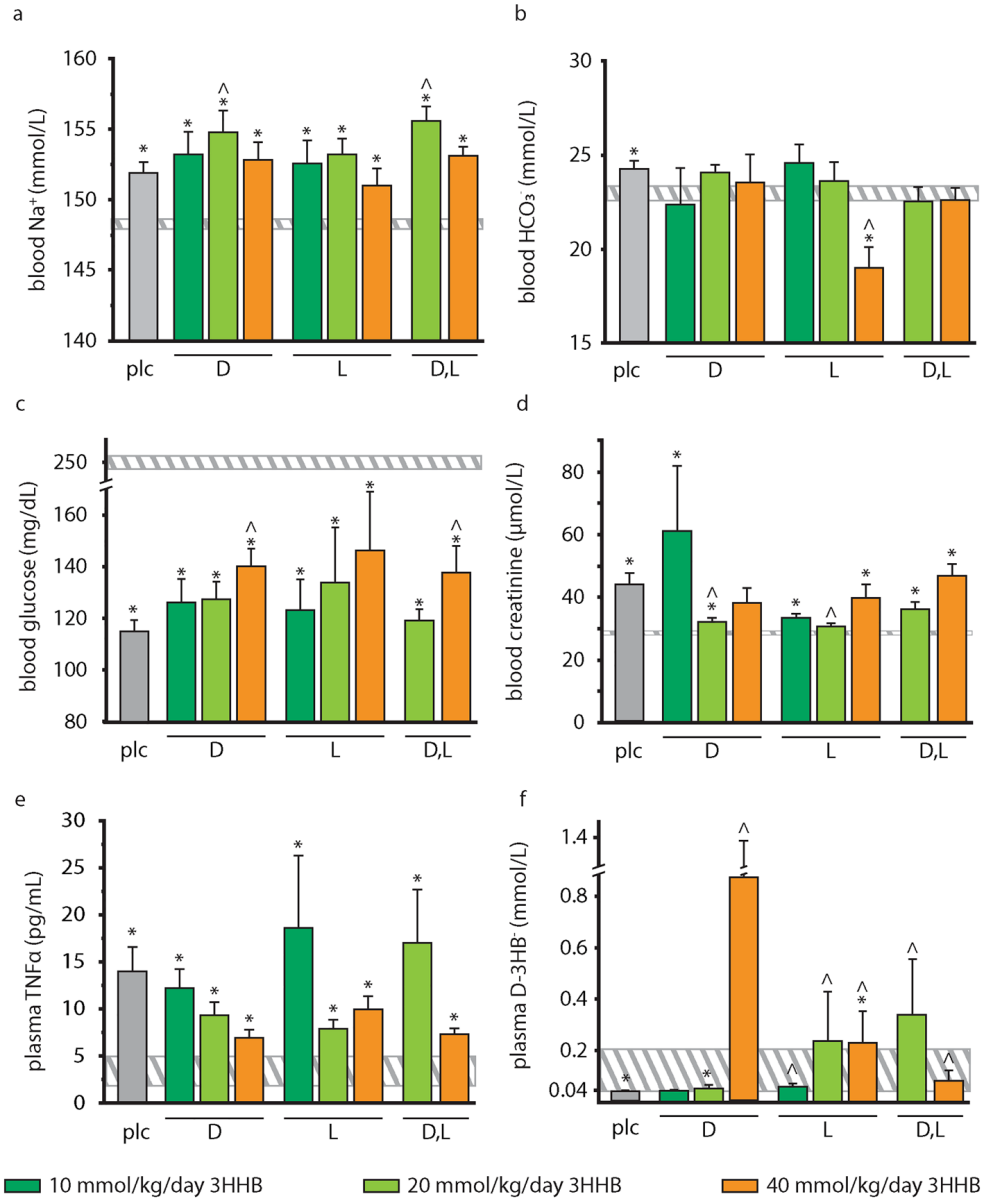
Impact of increasing bolus doses of pure and mixed racemic enantiomers of 3HHB on parameters of metabolism, organ function and inflammation. Whole blood taken at sacrifice was assessed for (**a**) blood Na^+^; (**b**) blood HCO_3_^−^; (**c**) blood glucose; (**d**) blood creatinine; (**e**) plasma TNFα; (**f**) plasma d-3HB^−^; Data are shown as mean ± standard error of the mean. Horizontal bars: healthy control (n = 80); grey, vertical bars: placebo (n = 74); d-3HHB: 10 mmol/kg/day (n = 18); 20 mmol/kg/day (n = 19); 40 mmol/kg/day (n = 16); l-3HHB: 10 mmol/kg/day (n = 15); 20 mmol/kg/day (n = 19); 40 mmol/kg/day (n = 14); d,l-3HHB: 20 mmol/kg/day (n = 16); 40 mmol/kg/day (n = 17). *ctr* healthy control, *plc* placebo, *3HHB* 3-hydroxybutyl-3-hydroxybutanoate ester. *p ≤ 0.05 vs healthy control; ^^^p ≤ 0.05 vs placebo.

We compared pure enantiomers (R)-3-hydroxybutyl-(R)-3-hydroxybutanoate ester (further denoted as d-3HHB, Fig. 1a) and (S)-3-hydroxybutyl-(S)-3-hydroxybutanoate ester (further denoted as l-3HHB) or a 50/50 mixture of both pure enantiomers (further denoted as d,l-3HHB, Fig. 1a) with placebo treatment. While consecutively increasing the dose of the ketone esters in the septic mice, severity of illness scores and mortality were assessed up to 5 days of sepsis. As compared with placebo, doses of 10 and 20 mmol/kg/day pure d- or l-3HHB enantiomers or of 20 mmol/kg/day racemic d,l-3HHB mixture did not adversely affect severity of illness scores or mortality (Fig. 1b,c). At 40 mmol/kg/day, d-3HHB and d,l-3HHB also did not alter severity of illness scores and mortality as compared with placebo, whereas l-3HHB increased severity of illness scores (p = 0.01 vs. placebo) and mortality (p = 0.05 vs. placebo) (Fig. 1b,c Supplementary Fig. 1). At 80 mmol/kg/day, both pure and mixed d-HHB and l-HHB bolus injections were 100% lethal before day 5 of sepsis (p < 0.0001 vs placebo, Fig. 1b,c Supplementary Fig. 1).

### Effect of increasing bolus doses of pure and mixed racemic enantiomers of 3HHB on muscle weakness and muscle wasting

After 5 days of sepsis, at sacrifice, muscle force of the Extensor digitorum longus muscle (EDL) was determined ex vivo. Septic mice treated with the lowest tested dose of 10 mmol/kg/day of the pure d- or l-3HHB enantiomers displayed a similar decrease in muscle force as placebo-treated septic mice (Fig. 2a). In mice treated with 20 mmol/kg/day of l-3HHB, specific muscle force increased compared to placebo (p = 0.007), up to 93% of healthy control levels (Fig. 2a,b). Treatment with 20 mmol/kg/day of d-3HHB or d,l-3HHB did not affect specific muscle force compared to placebo treated mice, but 40 mmol/kg/day of d-3HHB (p = 0.05) or d,l-3HHB (p = 0.04) also increased specific muscle force as compared with placebo to respectively 93% and 90% of healthy control levels (Fig. 2a).

Tibialis anterior muscle mass and EDL muscle mass was reduced in all septic groups compared to healthy mice (p ≤ 0.0001, Fig. 2c,d). This loss of muscle mass was largely unaffected by ketone ester administration although 40 mmol/kg/day of d,l-3HHB and l-3HHB attenuated the loss in Tibialis muscle mass (p = 0.01 compared to placebo) and EDL muscle mass respectively (p = 0.01 compared to placebo). In addition, muscle gene expression levels of atrophy markers *Trim63* and *Fbxo32* were equally increased in all septic groups (p ≥ 0.1 vs placebo) as compared with healthy control mice (p ≤ 0.0001, Fig. 2e,f).

### Effect of increasing bolus doses of pure and mixed racemic enantiomers of 3HHB on parameters of metabolism and inflammation

Plasma parameters of metabolism and inflammation were quantified after 5 days of sepsis, at sacrifice. As compared with placebo, the 20 mmol/kg/day of d- and d,l-3HHB evoked a moderate further increase in blood Na^+^ levels (p ≤ 0.02, Fig. 3a). In contrast, blood HCO3^−^ was decreased by 40 mmol/kg/day of l-3HHB (p ≤ 0.0001 vs placebo, Fig. 3b). At 40 mmol/kg/day, d- and d,l-3HHB bolus injection resulted in moderately higher blood glucose concentrations in the septic mice as compared with placebo (p ≤ 0.03), whereas the other 3HHB bolus formulations resulted in similar blood glucose levels (p ≥ 0.1 vs placebo, Fig. 3c). Blood creatinine levels were overall increased in septic mice, albeit less than in the placebo group, in the groups receiving 20 mmol/kg/day of d- and l-3HHB supplementation (p ≤ 0.03 vs placebo, Fig. 3d). Plasma TNFα was increased by sepsis in all groups (p ≤ 0.0001, vs healthy controls, Fig. 3e) similar to placebo (p ≥ 0.07, Fig. 3e).

After 5 days of sepsis, also plasma 3HB was quantified at sacrifice, which was 4 h after the last bolus dose of 3HHB. Only d-3HB was measured due to the stereoselective nature of the used enzymatic assay. The plasma value of d-3HB thus represents the net result of endogenous 3HB production and metabolism and metabolism of exogenous d-3HHB. As compared with placebo, daily supplementation with 3HHB evoked increased plasma d-3HB concentrations (p ≤ 0.05), except for the 10 and 20 mmol/kg/day of d-3HHB (p ≥ 0.2, Fig. 3f).

### Effect of increasing bolus doses of pure and mixed racemic enantiomers of 3HHB on plasma and hepatic lipid markers

**Figure 4:**
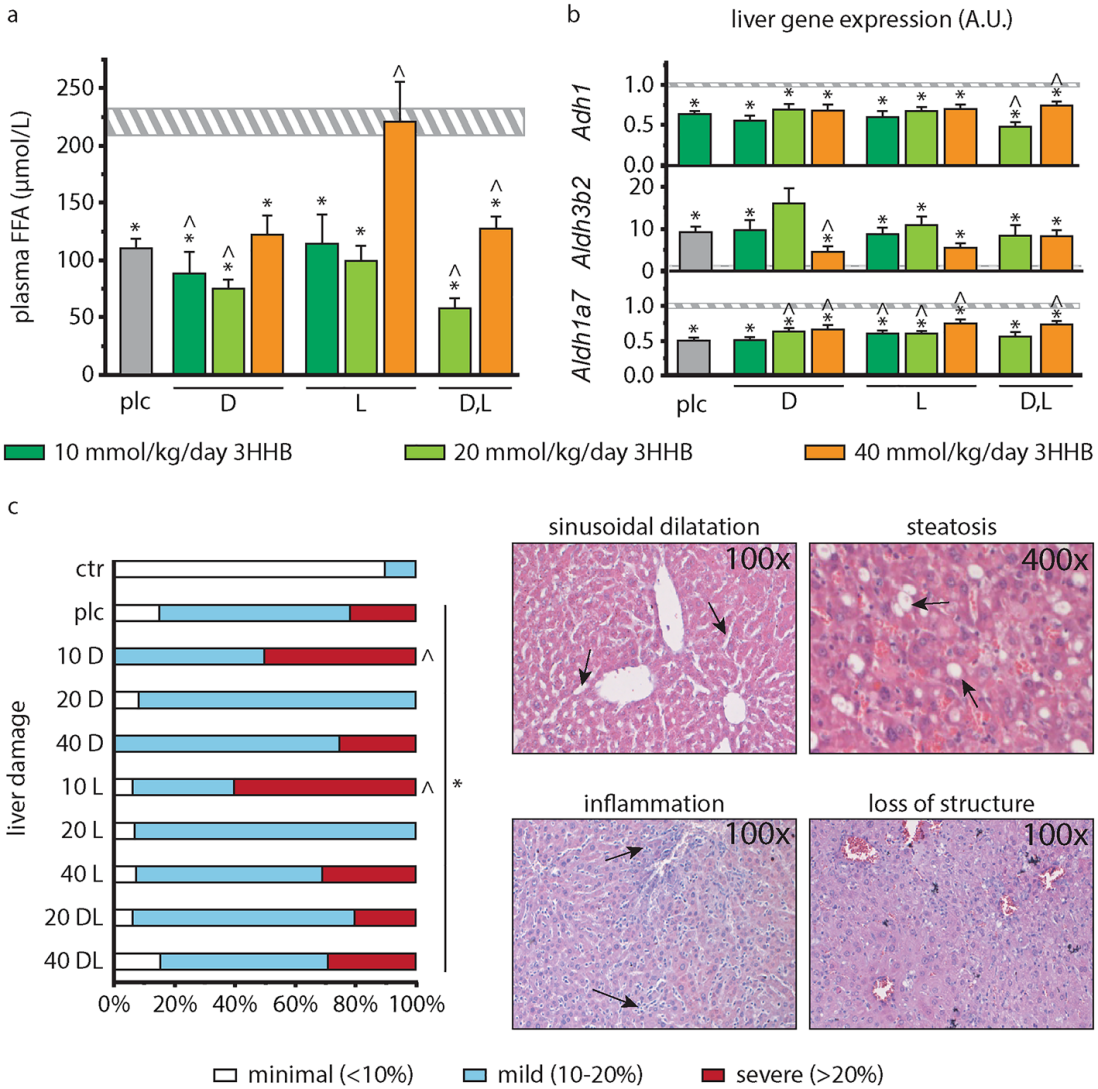
Potential toxicity of increasing bolus doses of pure and mixed racemic enantiomers of 3HHB and impact on markers of the hepatic lipid profile. (**a**) Plasma free fatty acid concentration. (**b**) Relative mRNA expression of liver alcohol metabolism markers *Adh1*, *Aldh3b2* and *Aldh1a7*. Gene expression data are normalized to *Rn18s* and were expressed as a fold change of the mean of control mice. (**a**,**b**) Horizontal bar: healthy control (n = 80) grey, vertical bars: placebo (n = 74); d-3HHB: 10 mmol/kg/day (n = 18); 20 mmol/kg/day (n = 19); 40 mmol/kg/day (n = 16); l-3HHB: 10 mmol/kg/day (n = 15); 20 mmol/kg/day (n = 19); 40 mmol/kg/day (n = 14); d,l-3HHB: 20 mmol/kg/day (n = 16); 40 mmol/kg/day (n = 17). (**c**) Semi-quantitative histological assessment of liver damage. Data are shown as cumulative percentages of the respective group. ctr (n = 79), plc (n = 65), d-3HHB: 10 mmol/kg/day (10D, n = 18); 20 mmol/kg/day (20D, n = 12); 40 mmol/kg/day (40D n = 16); L-3HHB: 10 mmol/kg/day (10L, n = 15); 20 mmol/kg/day (20L, n = 14); 40 mmol/kg/day (40L, n = 13); d,l-3HHB: 20 mmol/kg/day (20DL, n = 15); 40 mmol/kg/day (40DL, n = 17). (**a–c**) *ctr* healthy control, *plc* placebo, *3HHB* 3-hydroxybutyl-3-hydroxybutanoate ester. *p ≤ 0.05 vs healthy control; ^^^p ≤ 0.05 vs placebo.

In the metabolism of 3HHB to 3HB^−^ also 1,3-butanediol is formed which can be dehydrogenized to 3HB^−^ but also to lipid compounds. As compared with placebo, plasma free fatty acid concentrations were further reduced by 10 or 20 mmol/kg/day of D-3HHB (p ≤ 0.05), and by 20 mmol/kg/day d,l-3HHB (p = 0.002, Fig. 4a). However, at a dose of 40 mmol/kg/day, both pure and mixed racemic 3HHB enantiomers increased plasma free fatty acid concentrations, even up to healthy control levels in the group receiving l-3HHB (Fig. 4a). The effect on plasma free fatty acids was not accompanied with an effect on liver triglyceride content, which was similar for all septic groups (p ≥ 0.1 vs placebo, Supplementary Fig. 2). Hepatic gene expression of *Adh1* and *Aldh3b2*, involved in the metabolism of 1,3-butanediol, was decreased by sepsis and largely unaffected by 3HHB treatment (Fig. 4b). Only the racemic d,l-3HHB mixture evoked decreased *Adh1* gene expression at a dose of 20 mmol/kg/day and increased expression at a dose of 40 mmol/kg/day (p ≤ 0.03 vs placebo). At 40 mmol/kg/day of D-3HHB, *Aldh3b2* gene expression was lower than with placebo (p = 0.02, Fig. 4b). Hepatic gene expression levels of *Aldh1a7* was also reduced by sepsis but increased by all 3HHB formulations as compared with placebo (p ≤ 0.04), except for 10 mmol/kg/day of d-ester and 20 mmol/kg/day of d,l-ester (p ≥ 0.5 vs placebo, Fig. 4d).

Plasma GGT, a hepatic marker indicative for alcohol-induced damage, was similar in healthy controls, placebo-treated septic mice or mice receiving pure or mixed racemic 40 mmol/kg/day 3HHB (p ≥ 0.4, data not shown). Liver histology showed a similar increase in liver damage score in all septic groups (p < 0.0001 vs healthy controls), not affected by any of the tested 3HHB doses (p ≥ 0.07 vs placebo), except for groups receiving 10 mmol/kg/day of d- and l-3HHB (p ≤ 0.02 vs placebo, Fig. 4c).

### Effect of sepsis on estimated 3HB clearance after bolus injection of 3HHB

**Figure 5:**
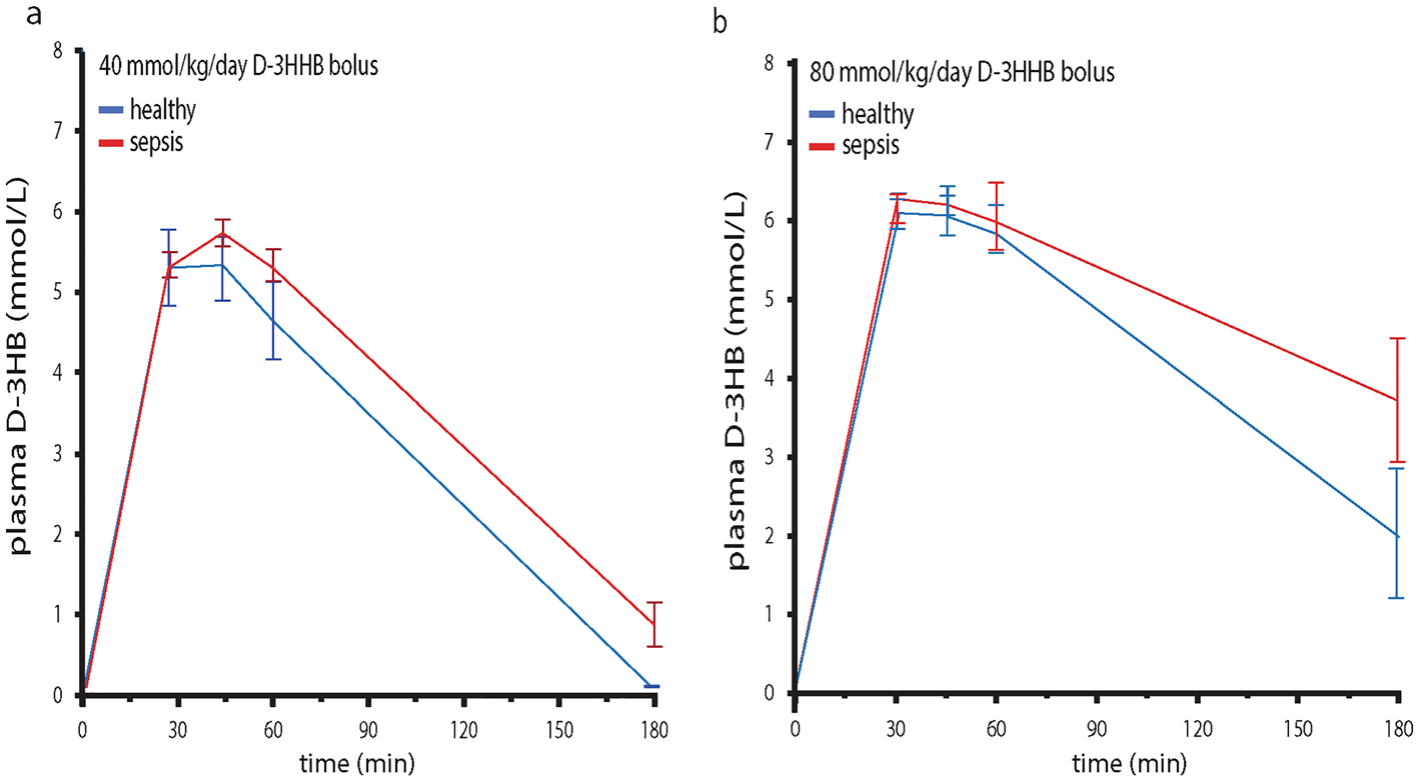
Impact of sepsis on estimated d-3HB clearance after bolus injection of d-3HHB. Repeated blood samples were collected and d-3HB was quantified at 30, 45, 60 and 180 min after bolus injection of pure d-3HHB at (**a**) 40 mmol/kg/day or (**b**) 80 mmol/kg/day. Healthy mice are indicated with blue lines, septic mice are indicated with red lines. N = 27.

To assess whether the metabolically challenging condition of sepsis affected occurrence and clearance of 3HB^−^ after bolus injection of 3HHB, plasma 3HB concentration was quantified at 30, 45, 60 and 180 min after bolus administration in healthy and septic mice. As only d-3HB could be measured due to the stereoselective nature of the enzymatic assay, only pure d-3HHB was tested. At a dose of 40 mmol/kg/day of d-3HHB bolus, plasma d-3HB increased to higher concentrations and was cleared slower in septic mice as compared with healthy mice (p = 0.04, Fig. 5a). After a bolus injection of the 80 mmol/kg/day dose, neither healthy nor septic mice were able to completely clear the bolus-induced rise in plasma d-3HB after 180 min, but septic mice showed again a slower clearance as compared with healthy mice (p = 0.02, Fig. 5b).

### Effect of a continuous infusion of 3HHB esters on severity of illness scores, mortality and muscle force

**Figure 6:**
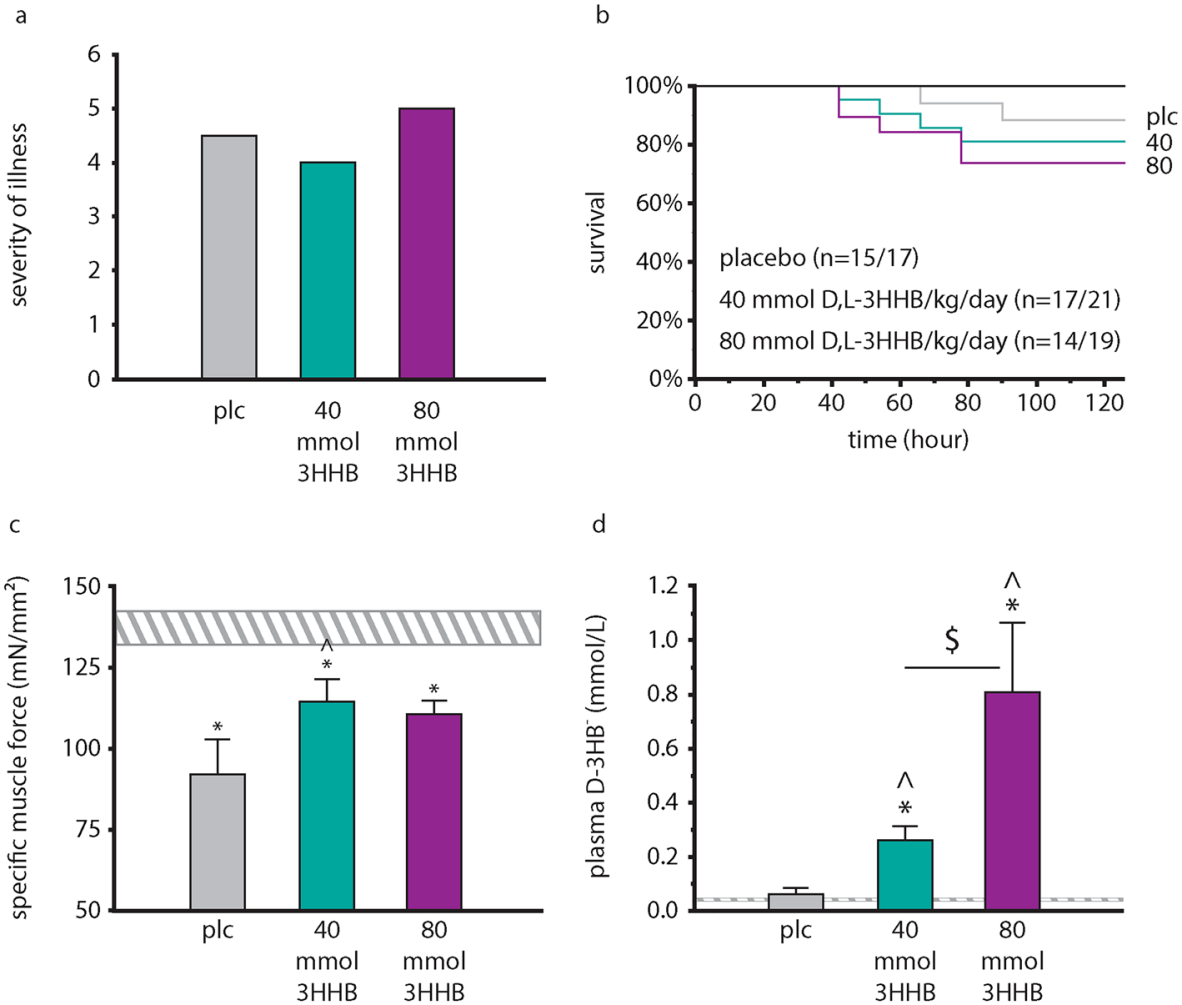
Impact of continuous infusion of d,l-3HHB on severity of illness and mortality, muscle force and circulating ketone level. (**a**) Cumulative severity of illness. Data are shown as medians. Number of animals equals total number of animals reported in panel (**b**). (**b**) Cumulative survival of the study. Number of animals is reported as n = survivors/total. 40: 40 mmol/kg/day d,l-3HHB; 80: 80 mmol/kg/day d,l-3HHB. (**c**) Ex vivo EDL force measurements presented as peak tetanic tension per unit muscle mass. (**d**) Plasma d-3HB^−^ levels assessed at sacrifice. (**c**,**d**) Data are shown as mean ± standard error of the mean. White–grey shaded horizontal bar, healthy control (n = 15); vertical bars: plc (n = 15); d,l-3HHB: 40 mmol/kg/day (n = 17); 80 mmol/kg/day (n = 14). *Ctr* healthy control, *plc* placebo, *3HHB* 3-hydroxybutyl-3-hydroxybutanoate ester. *p ≤ 0.05 vs healthy control; ^^^p ≤ 0.05 vs placebo; ^$^p ≤ 0.05 between groups.

We next tested in a third animal experiment whether the observed toxicity of 3HHB ester by bolus injections at 80 mmol/kg/day could be avoided by administering d,l-3HHB ester as a continuous infusion, while remaining effective in improving muscle force. In contrast to the bolus supplementation, similar severity of illness scores (p ≥ 0.7) and mortality (p ≥ 0.2) were observed for 40 and 80 mmol/kg/day d,l-3HHB-continuous as compared with placebo (Fig. 6a,b). Furthermore, as compared with placebo, the d,l-3HHB-continuous infusion increased specific muscle force at 40 mmol/kg/day (p = 0.03, Fig. 6c), and tended to increase specific muscle force at 80 mmol/kg/day (p = 0.08, Fig. 6c). As was observed with bolus injection, muscle mass of Tibialis anterior and EDL was reduced by sepsis and unaffected by 3HHB continuous infusion (p ≤ 0.0001 vs healthy controls, Supplementary Fig. 3).

After 5 days, a dose-dependent increase in plasma d-3HB concentration was observed with 40 and 80 mmol/kg/day d,l-3HHB-continuous infusion as compared with healthy controls and with placebo-treated septic mice (p ≤ 0.0001, Fig. 6d). As compared with placebo, blood Na^+^, HCO3^−^, glucose and creatinine concentrations were not affected by continuous d,l-3HHB infusion, for both doses tested (p ≥ 0.2, Supplementary Fig. 4).

## Discussion

In a mouse model of sepsis-induced prolonged critical illness, treatment with bolus injections of the ketone ester 3HHB was able to partially prevent muscle weakness but not wasting. The l-enantiomer bolus reached maximal muscle protection at 20 mmol/kg/day but became toxic at 40 mmol/kg/day. The bolus of the d-enantiomer and of the racemic mixture evoked maximal protection against muscle weakness at 40 mmol/kg/day without signs of toxicity. However, at a bolus dose of 80 mmol/kg/day, either pure or mixed racemic 3HHB was lethal. Sepsis impaired plasma clearance of 3HB^−^ after bolus injection causing prolonged hyperketonemia. In contrast, when the racemic 3HHB mixture was continuously infused, either at 40 or 80 mmol/kg/day, bolus injection toxicity was avoided and muscle weakness was as effectively prevented at 40 mmol/kg/day, as with bolus injections.

Similar to the earlier observed effect of the ketone salt 3HB-Na^8^, also the ketone ester 3HHB was able to partly protect against the development of muscle weakness in septic mice. The sepsis-induced sustained loss in muscle mass and upregulation of markers of muscle atrophy were not affected by 3HHB supplementation, similar to the effects of 3HB-Na^8^. These and previous data point to a role for 3HB as a signaling molecule rather than being used as an alternative energy source in the context of critical illness^8,9^. Similarly, during LPS-induced inflammation in humans, ketone salts were not able to counteract protein breakdown^24^. This is different from studies performed in healthy volunteers and in cancer cachexia, where an anticatabolic effect of ketones was observed^13,25–28^.

The racemic mixture of 3HHB reached a maximal effect on muscle force at 40 mmol/kg/day, the same dose at which 3HB-Na reached its maximal effect^10^. However, as one molecule of 3HHB can be metabolized to two molecules of 3HB, this difference in efficacy might point to an incomplete metabolism of 3HHB. Indeed, 3HHB is expected to undergo hydrolysis to 3HB^-^ and 1,3-butanediol by carboxylesterases, present in blood and liver, followed by metabolism of 1,3-butanediol to 3HB^−^ in the liver by alcohol and aldehyde dehydrogenases^18,29^. A potentially suppressed conversion is supported by the finding that alcohol and aldehyde dehydrogenases *Adh1* and *Aldh1a7* gene expression was strongly suppressed by sepsis, although *Aldh3b2* expression was increased. Of note, as GGT was unaffected by the ester treatment, and no additional histological liver damage was observed, overt toxic side effects of potentially incomplete 1,3-butanediol conversion seem unlikely.

Importantly, the protective effect on muscle force was not specific for one enantiomer as both l-3HHB and d-3HHB were able to improve muscle force. Although d-3HB is the predominant form, also l-3HB is endogenously produced^20,30^. In our experiments, the L-enantiomer reached its maximal protective effect on muscle force at a lower dose than the d-enantiomer, but also caused toxic side effects at a lower dose than the d-ester or the racemic mixture. This difference in efficacy and toxicity might be caused by a difference in enantiomer ester metabolism. A study in perfused rat livers indicated that where d-butanediol was mostly converted to d-3HB^−^, a large part of l-butanediol was converted to lipids and CO_2_^18^. In vivo administration of l-butanediol increased plasma levels of cholesterol, triglycerides and free fatty acids^31^. Endogenously, l-3HB^−^ is more favorably used for the synthesis of sterols and fatty acids, whereas d-3HB^−^ is more favorably used in the TCA cycle^16^. We previously documented that the protective effect of 3HB-Na was associated with an increased conversion to cholesterol rather than it being used in the TCA cycle^8,9^, which fits with the preferred fate of the l-enantiomer. The observed increase in plasma free fatty acids in the group receiving 40 mmol/kg/day l-3HHB further supports a difference in metabolism between enantiomers.

At doses up to 40 mmol/kg/day, and unlike the ketone salt 3HB-Na, ketone ester injections did not disturb blood Na^+^, bicarbonate or creatinine concentrations in mice surviving up to day 5 of sepsis. Also, no additional histological liver damage or hepatic triglyceride accumulation was observed with the ester treatment. Plasma TNFα was also not affected by 3HHB treatment, in contrast to earlier described attenuation of pro-inflammatory cytokines in LPS-treated cells^32^ and in rats^33^. Of note, in healthy conditions plasma glucose and free fatty acids typically decrease upon administration of exogenous ketones^34,35^, an effect that was not that clearly visible in this setting of critical illness. However, also during health, these effects are often short lasting, with both glucose and fatty acids recurring to normal levels within hours^36,37^ and even unaffected levels with prolonged administration of exogenous ketone esters in rodents and humans^12,38,39^. Furthermore, critical illness by itself already strongly affects glucose and lipid homeostasis^40,41^, which might have interfered with the normal actions of 3HB. In addition, the known direct inhibitory effect on lipolysis of 3HB^34^ might have been counteracted by an increased conversion of l-1,3-butanediol to fatty acids^18^.

We observed 100% lethality with bolus injections of pure and racemic 3HHB at a dose of 80 mmol/kg/day. In contrast, toxicity studies of oral administration of d-3HHB performed in mice, rats and humans, showed safety of the doses that we tested in our experiments. Mice receiving an oral diet containing 91 mmol/kg/day D-3HHB for 8 months did not suffer from toxic side effects^39^. In rats, a 28 days diet containing d-3HHB at 85.1 mmol/kg/day was not accompanied by changes indicative of toxicity^38^. In humans, the oral administration of d-3HHB was tested and found safe up to a daily dose of 12 mmol/kg/day^11,29^. Of note, the human equivalent daily dose is estimated to be 12.3 times lower than that of mice^42^, which means that the toxic dose in septic mice of 80 mmol/kg/day would correspond to a human dose of approximately 6.5 mmol/kg/day. The pharmacokinetic analysis indicated that septic animals had an impaired ability to clear 3HB^−^ after a bolus injection. Critical illness is indeed associated with impaired cellular bioenergetics and mitochondrial function which might have affected cellular metabolism of 3HHB and 3HB^43–45^. In addition, when plasma 3HB^−^ levels surpasses the buffering capacity of the body, this may result in lethal metabolic acidosis^46^. This risk is likely higher in the context of critical illness, as such patients suffer from metabolic and electrolyte disturbances, but also from endothelial dysfunction and organ failure^47,48^. In contrast to the bolus injection, a continuous infusion of 80 mmol/kg/day appeared without toxic side effects, indicating that indeed the bolus injection and not the daily dose was the culprit.

Our study has two important limitations to highlight. First, despite the clinical relevance and earlier validation of our mouse model of sepsis-induced, fluid-resuscitated, antibiotics- and analgesia-treated and parenterally fed critical illness, any translation of results to the human clinical setting should be done with caution. Second, we did not determine plasma or urine concentrations of 3HHB enantiomers and of their chiral metabolites 3HB and 1,3-butanediol with mass spectrometry. Only d-3HB plasma was determined by enzymatic analysis, which limits the conclusions on pharmacokinetics of the ester enantiomers and their metabolites. A strength of our study is the used delivery route—where numerous studies with orally administered ketone esters in rodents and humans in different settings and disease models are available, information on the parenteral administration of ketones and ketone esters is scarce^37^. We here studied subcutaneous bolus injections and intravenous continuous infusion in the context of critical illness and found a clear safety benefit with the latter method. In addition, we provided insights in the efficacy and toxicity of both enantiomers. Indeed, although l-3HHB, d-3HHB and the racemic mixture were all able to protect against muscle weakness, the l-enantiomer had a different metabolic profile and a higher toxicity risk, which suggests that the d-3HHB monoester is likely the preferred choice for future translational studies.

## Conclusion

Treatment of septic mice with pure and racemic mixtures of the ketone ester 3HHB partly prevented muscle weakness. Doubling the effective ester bolus dose to 80 mmol/kg/day in septic mice was lethal, whereas this toxicity was completely avoided by continuous infusion of the same dose. Whether continuous infusion of ketone esters represents a promising intervention to also prevent ICU-acquired weakness in human patients should be investigated.

## Methods

### Animal studies: experimental setup

We used a validated, centrally catheterized, fluid-resuscitated 5-day model of cecal ligation and puncture-induced prolonged sepsis, as previously described^49^. Septic mice were treated with antibiotics and analgesics twice daily and received continuous fluid resuscitation with a mixture of balanced colloids and crystalloids for 20 h, followed by continuous intravenous infusion of standard mixed PN (Olimel N7E, Baxter, Lessines, Belgium) at 5.8 kcal/day. More details can be found in the online supplement. All animals were treated according to the principles of Laboratory Animal Care (U.S. national Society for Medical Research) and the Guide for Care and Use of Laboratory Animals (National Institutes of Health). The Institutional Ethical Committee for Animal Experimentation of the KU Leuven had approved the protocols for these animal studies. The study is in accordance with the ARRIVE guidelines.

#### Experiment 1: Effect of increasing bolus doses of ketone esters on muscle force, morbidity and mortality

Parenterally fed septic mice received, from day 1 onwards, twice daily subcutaneous bolus injections of enantiomers (R)-3-hydroxybutyl-(R)-3-hydroxybutanoate ester (d-3HHB) and (S)-3-hydroxybutyl-(S)-3-hydroxybutanoate ester (l-3HHB) or a 50/50 mixture of both pure enantiomers (d,l-3HHB). d-3HHB (manufactured by Innov’Orga, Reims, France) and l-3HHB (manufactured by Synthenova, Herouville Saint Clair, France) were dissolved in Plasmalyte A Viaflo (Baxter) at the appropriate concentrations. Per chiral form, alone or combined, a stepwise dose increase was tested, as described in the online supplement, starting with the group receiving a dose of 10 mmol/kg/day, followed by 20 mmol/kg/day, 40 mmol/kg/day and 80 mmol/kg/day. Placebo was an isovolumetric dose of glucose (187.5 mg/day), also dissolved in Plasmalyte. Pain/discomfort was assessed twice daily by means of the Mouse Grimace Score^19^, and the summed score was used as a severity of illness score. Non-survivors were allocated the maximum severity of illness score + 1. The doses were increased until toxicity (increased severity of illness or lethality) could be observed. In the absence of toxicity, for each dose-step the experiment was continued until 14 animals survived up to day 5 from which ex vivo muscle force measurements could be obtained (more details below). This sample number allowed to detect with a similar effect size as with ketone salts^8^, an improvement in specific muscle force with an a-error of 0.05 and 80% power (calculated with two-sided t-test). For each dose-step a group of control animals and a group of placebo-treated mice were included.

#### Experiment 2: Pharmacokinetic study

To document the peak plasma concentration and plasma clearance of 3HB^-^ after bolus injections of the ketone esters, healthy mice and septic mice on day 2 of sepsis received d-3HHB injection (40 or 80 mmol/kg/day) after which blood samples were collected at 30 min, 45 min, 60 min and 180 min. For each concentration the experiment was continued until at least 5 animals were included.

#### Experiment 3: Effect of continuous infusion of ketone esters on muscle force, morbidity and mortality

This third experiment was designed to test whether the observed toxicity of 3HHB by a bolus injection at 80 mmol/kg/day (experiment 1) could be avoided by administering 3HHB as a continuous infusion, while remaining effective in improving muscle force. Only the racemic mixture was tested as it combined the potential beneficial and toxic effects of the pure enantiomers: l-3HHB had a higher efficacy, but d-3HHB had a lower toxicity when administered as a bolus. To assess efficacy of a continuous administration of d,l-3HHB, septic mice received PN (0.2 mL/h) supplemented with a racemic mixture of 40 mmol or 80 mmol/kg/day d,l-3HHB (0.033 mL/h) from day 1 onwards, dissolved in Plasmalyte and administered through an Octopus catheter (Vygon, Écouen, France) with non-return valves to prevent backtracking of fluids when infusing multiple fluids simultaneously. In the absence of toxicity, similar in setup as experiment 1, the experiment was continued until 14 animals survived up to day 5 from which ex vivo muscle force measurements could be obtained.

### Ex vivo measurement of muscle force

After 5 days of sepsis, the timeframe required to develop sepsis-induced muscle weakness^50^, immediately after euthanasia, the EDL muscle was isolated and suspended in a temperature controlled and continuously perfused organ bath^8^. The maximal isometric tetanic force was measured by tetanic stimuli and the specific maximal isometric tetanic force was determined according the muscle cross-sectional area. Data collection was done with use of the Dynamic Muscle Analysis software (Aurora scientific, Ontario, Canada). More details are available in the online supplement.

### Blood and plasma analyses

For 5-day survivors, blood Na^+^, HCO_3_^−^ and creatinine concentrations were measured at sacrifice with use of the Epoc^®^ Blood Analysis System (Siemens Healthineers, The Hague, The Netherlands). Plasma 3HB^−^ was quantified with an in-house developed enzymatic assay^6^. Impact of 3HHB on inflammation was assessed by plasma TNFα quantification (Mouse TNF-alpha Quantikine HS ELISA Kit, R&D systems, Minneapolis, MN, USA). Potentially toxic impact of hepatic 3HHB metabolism, which can be evoked by intermediate formation of 1,3-butanediol, was assessed by measuring plasma gamma-glutamyltransferase (GGT, MAK090, Sigma Aldrich, Saint-Louis, MO, USA).

### Tissue analyses

To determine markers of 3HHB metabolism, inflammation and tissue damage in 5-day survivors, RNA was extracted from liver and muscle samples and reverse-transcribed. Real-time quantification of cDNA was performed using commercially available TaqMan assays (Supplementary Table 1). Data are shown normalized to 18S ribosomal RNA (*Rn18s*) and were expressed as a fold change of the mean of control mice. To assess histological damage in liver, for 5-day survivors, hematoxylin and eosin stained formalin fixed paraffin liver sections were semi-quantitatively assessed for changes in lipid accumulation, infiltration of inflammatory cells, sinusoidal dilatation and loss of structure^51^. These scores were combined into an overall damage score further explained in the online supplement. Hepatic triglyceride content was determined with a commercially available colorimetric assay (triglyceride quantification kit, Abcam, Cambridge, UK).

### Statistics

All analyses were performed with JMP^®^ Pro 15 (SAS Institute Inc., Cary, NC, USA). Continuous normally distributed data were compared with one-way analysis of variance (ANOVA), Post hoc Fisher's LSD test (Student’s t Test) were used for multiple comparisons, where necessary, after log- or (double) square root-transformation to obtain a near-normal distribution. Not-normally distributed data were analyzed with non-parametric Wilcoxon tests. Mortality rates were compared with Chi-square tests. Pharmacokinetic profiles over time were compared with multivariate analysis of variance (MANOVA). No corrections for multiple comparisons were performed.

### Ethical approval and consent to participate

The Institutional Ethical Committee for Animal Experimentation of the KU Leuven had approved the protocols for these animal studies (internal project numbers P048/2019 and P135/2020).

## Supplementary Information

Supplementary Information.

## Data Availability

The datasets used during the current study are available from the corresponding author on reasonable request.

## Abbreviations

3HB: 3-Hydroxybutyrate
3HB-Na: D,l-3-Hydroxybutyrate sodium salt
3HHB: 3-Hydroxybutyl-3-hydroxybutanoate
ADH1: Alcohol dehydrogenase 1 (class I)
ALDH1A7: Aldehyde dehydrogenase family 1, subfamily A7
ALDH3B2: Aldehyde dehydrogenase 3 family, member B2
ANOVA: One-way analysis of variance
CSA: Cross sectional area
EDL: Extensor digitorum longus
FBXO32: F-box protein 32
GGT: Gamma-glutamyltransferase
ICU: Intensive care unit
LPS: Lipopolysaccharide
PN: Parenteral nutrition
RN18S: 18S ribosomal RNA
TCA: Tricarboxylic acid
TNFα: Tumor necrosis factor alpha
TRIM63: Tripartite motif-containing 63

## References

[1] Vanhorebeek I, Latronico N, Van den Berghe G (2020). ICU-acquired weakness. Intensive Care Med..

[2] Puthucheary Z, Harridge S, Hart N (2010). Skeletal muscle dysfunction in critical care: Wasting, weakness, and rehabilitation strategies. Crit. Care Med..

[3] Van Aerde N, Meersseman P, Debaveye Y, Wilmer A, Gunst J, Casaer MP (2020). Five-year impact of ICU-acquired neuromuscular complications: A prospective, observational study. Intensive Care Med..

[4] Casaer MP, Langouche L, Coudyzer W, Vanbeckevoort D, De Dobbelaer B, Guiza FG (2013). Impact of early parenteral nutrition on muscle and adipose tissue compartments during critical illness. Crit. Care Med..

[5] Hermans G, Casaer MP, Clerckx B, Guiza F, Vanhullebusch T, Derde S (2013). Effect of tolerating macronutrient deficit on the development of intensive-care unit acquired weakness: A subanalysis of the EPaNIC trial. Lancet Respir. Med..

[6] De Bruyn A, Gunst J, Goossens C, Vander Perre S, Guerra GG, Verbruggen S (2020). Effect of withholding early parenteral nutrition in PICU on ketogenesis as potential mediator of its outcome benefit. Crit. Care..

[7] De Bruyn A, Langouche L, Vander Perre S, Gunst J, Van den Berghe G (2021). Impact of withholding early parenteral nutrition in adult critically ill patients on ketogenesis in relation to outcome. Crit. Care..

[8] Goossens C, Weckx R, Derde S, Dufour T, Vander Perre S, Pauwels L (2019). Adipose tissue protects against sepsis-induced muscle weakness in mice: From lipolysis to ketones. Crit. Care..

[9] Goossens C, Weckx R, Derde S, Vander Perre S, Derese I, Van Veldhoven PP (2021). Altered cholesterol homeostasis in critical illness-induced muscle weakness: Effect of exogenous 3-hydroxybutyrate. Crit. Care..

[10] Weckx R, Goossens C, Derde S, Pauwels L, Vander Perre S, Van den Berghe G (2021). Identification of the toxic threshold of 3-hydroxybutyrate-sodium supplementation in septic mice. BMC Pharmacol. Toxicol..

[11] Shivva V, Cox PJ, Clarke K, Veech RL, Tucker IG, Duffull SB (2016). The population pharmacokinetics of D-beta-hydroxybutyrate following administration of (R)-3-hydroxybutyl (R)-3-hydroxybutyrate. AAPS J..

[12] Soto-Mota A, Vansant H, Evans RD, Clarke K (2019). Safety and tolerability of sustained exogenous ketosis using ketone monoester drinks for 28 days in healthy adults. Regul. Toxicol. Pharmacol..

[13] Cox PJ, Kirk T, Ashmore T, Willerton K, Evans R, Smith A (2016). Nutritional ketosis alters fuel preference and thereby endurance performance in athletes. Cell Metab..

[14] Poffe C, Ramaekers M, Van Thienen R, Hespel P (2019). Ketone ester supplementation blunts overreaching symptoms during endurance training overload. J. Physiol..

[15] Dearlove DJ, Faull OK, Clarke K (2019). Context is key: Exogenous ketosis and athletic performance. Curr. Opin. Physiol..

[16] Webber RJ, Edmond J (1977). Utilization of L(+)-3-hydroxybutyrate, D(-)-3-hydroxybutyrate, acetoacetate, and glucose for respiration and lipid synthesis in the 18-day-old rat. J. Biol. Chem..

[17] Tsai YC, Chou YC, Wu AB, Hu CM, Chen CY, Chen FA (2006). Stereoselective effects of 3-hydroxybutyrate on glucose utilization of rat cardiomyocytes. Life Sci..

[18] Desrochers S, David F, Garneau M, Jette M, Brunengraber H (1992). Metabolism of R- and S-1,3-butanediol in perfused livers from meal-fed and starved rats. Biochem. J..

[19] van Rijt WJ, Van Hove JLK, Vaz FM, Havinga R, Allersma DP, Zijp TR (2021). Enantiomer-specific pharmacokinetics of d,l-3-hydroxybutyrate: Implications for the treatment of multiple acyl-CoA dehydrogenase deficiency. J. Inherit. Metab. Dis..

[20] Tsai YC, Liao TH, Lee JA (2003). Identification of L-3-hydroxybutyrate as an original ketone body in rat serum by column-switching high-performance liquid chromatography and fluorescence derivatization. Anal. Biochem..

[21] Van Hove JL, Grunewald S, Jaeken J, Demaerel P, Declercq PE, Bourdoux P (2003). D,L-3-hydroxybutyrate treatment of multiple acyl-CoA dehydrogenase deficiency (MADD). Lancet.

[22] Jensen NJ, Nilsson M, Ingerslev JS, Olsen DA, Fenger M, Svart M (2020). Effects of beta-hydroxybutyrate on cognition in patients with type 2 diabetes. Eur. J. Endocrinol..

[23] Reintam Blaser A, Preiser JC, Fruhwald S, Wilmer A, Wernerman J, Benstoem C (2020). Gastrointestinal dysfunction in the critically ill: A systematic scoping review and research agenda proposed by the Section of Metabolism, Endocrinology and Nutrition of the European Society of Intensive Care Medicine. Crit. Care..

[24] Thomsen HH, Rittig N, Johannsen M, Moller AB, Jorgensen JO, Jessen N (2018). Effects of 3-hydroxybutyrate and free fatty acids on muscle protein kinetics and signaling during LPS-induced inflammation in humans: Anticatabolic impact of ketone bodies. Am. J. Clin. Nutr..

[25] Koutnik AP, Poff AM, Ward NP, DeBlasi JM, Soliven MA, Romero MA (2020). Ketone bodies attenuate wasting in models of atrophy. J. Cachexia Sarcopenia Muscle..

[26] Shukla SK, Gebregiworgis T, Purohit V, Chaika NV, Gunda V, Radhakrishnan P (2014). Metabolic reprogramming induced by ketone bodies diminishes pancreatic cancer cachexia. Cancer Metab..

[27] Nakamura K, Tonouchi H, Sasayama A, Ashida K (2018). A Ketogenic formula prevents tumor progression and cancer cachexia by attenuating systemic inflammation in colon 26 tumor-bearing mice. Nutrients.

[28] Nair KS, Welle SL, Halliday D, Campbell RG (1988). Effect of beta-hydroxybutyrate on whole-body leucine kinetics and fractional mixed skeletal muscle protein synthesis in humans. J. Clin. Investig..

[29] Clarke K, Tchabanenko K, Pawlosky R, Carter E, Todd King M, Musa-Veloso K (2012). Kinetics, safety and tolerability of (R)-3-hydroxybutyl (R)-3-hydroxybutyrate in healthy adult subjects. Regul. Toxicol. Pharmacol..

[30] Hsu WY, Kuo CY, Fukushima T, Imai K, Chen CM, Lin PY (2011). Enantioselective determination of 3-hydroxybutyrate in the tissues of normal and streptozotocin-induced diabetic rats of different ages. J. Chromatogr. B Anal. Technol. Biomed. Life Sci..

[31] Meenakshi C, Kumari KL, Devi CS (1995). Biochemical studies on the effect of S-1,3-butanediol of diabetes induced rats. Indian J. Physiol. Pharmacol..

[32] Fu SP, Li SN, Wang JF, Li Y, Xie SS, Xue WJ (2014). BHBA suppresses LPS-induced inflammation in BV-2 cells by inhibiting NF-kappaB activation. Mediat. Inflamm..

[33] Yamanashi T, Iwata M, Shibushita M, Tsunetomi K, Nagata M, Kajitani N (2020). Beta-hydroxybutyrate, an endogenous NLRP3 inflammasome inhibitor, attenuates anxiety-related behavior in a rodent post-traumatic stress disorder model. Sci. Rep..

[34] Taggart AK, Kero J, Gan X, Cai TQ, Cheng K, Ippolito M (2005). (D)-beta-Hydroxybutyrate inhibits adipocyte lipolysis via the nicotinic acid receptor PUMA-G. J. Biol. Chem..

[35] Balasse E, Ooms HA (1968). Changes in the concentrations of glucose, free fatty acids, insulin and ketone bodies in the blood during sodium beta-hydroxybutyrate infusions in man. Diabetologia.

[36] Stubbs BJ, Cox PJ, Evans RD, Santer P, Miller JJ, Faull OK (2017). On the metabolism of exogenous ketones in humans. Front. Physiol..

[37] White H, Heffernan AJ, Worrall S, Grunsfeld A, Thomas M (2021). A systematic review of intravenous beta-hydroxybutyrate use in humans—A promising future therapy?. Front. Med. (Lausanne)..

[38] Clarke K, Tchabanenko K, Pawlosky R, Carter E, Knight NS, Murray AJ (2012). Oral 28-day and developmental toxicity studies of (R)-3-hydroxybutyl (R)-3-hydroxybutyrate. Regul. Toxicol. Pharmacol..

[39] Kashiwaya Y, Bergman C, Lee JH, Wan R, King MT, Mughal MR (2013). A ketone ester diet exhibits anxiolytic and cognition-sparing properties, and lessens amyloid and tau pathologies in a mouse model of Alzheimer's disease. Neurobiol. Aging..

[40] Gunst J, De Bruyn A, Van den Berghe G (2019). Glucose control in the ICU. Curr. Opin. Anaesthesiol..

[41] Preiser JC, Ichai C, Orban JC, Groeneveld AB (2014). Metabolic response to the stress of critical illness. Br. J. Anaesth..

[42] Nair AB, Jacob S (2016). A simple practice guide for dose conversion between animals and human. J. Basic Clin. Pharm..

[43] Brealey D, Brand M, Hargreaves I, Heales S, Land J, Smolenski R (2002). Association between mitochondrial dysfunction and severity and outcome of septic shock. Lancet.

[44] McKenna HT, O'Brien KA, Fernandez BO, Minnion M, Tod A, McNally BD (2021). Divergent trajectories of cellular bioenergetics, intermediary metabolism and systemic redox status in survivors and non-survivors of critical illness. Redox Biol..

[45] Paumelle R, Haas JT, Hennuyer N, Bauge E, Deleye Y, Mesotten D (2019). Hepatic PPARalpha is critical in the metabolic adaptation to sepsis. J. Hepatol..

[46] Cahill GF, Veech RL (2003). Ketoacids? Good medicine?. Trans. Am. Clin. Climatol. Assoc..

[47] Lee JW (2010). Fluid and electrolyte disturbances in critically ill patients. Electrolyte Blood Press..

[48] Pool R, Gomez H, Kellum JA (2018). Mechanisms of organ dysfunction in sepsis. Crit. Care Clin..

[49] Derde S, Thiessen SE, Goossens C, Dufour T, Van den Berghe G, Langouche L (2017). Use of a central venous line for fluids, drugs and nutrient administration in a mouse model of critical illness. J. Vis. Exp..

[50] Goossens C, Marques MB, Derde S, Vander Perre S, Dufour T, Thiessen SE (2017). Premorbid obesity, but not nutrition, prevents critical illness-induced muscle wasting and weakness. J. Cachexia Sarcopenia Muscle..

[51] Jenniskens M, Guiza F, Oorts M, Perre SV, Derde S, Dufour T (2017). On the role of illness duration and nutrient restriction in cholestatic alterations that occur during critical illness. Shock.

