# Supplementary material for: Efficacy and safety of ketone ester infusion to prevent muscle weakness in a mouse model of sepsis-induced critical illness
Source: Sci Rep. 2022 Jun 22;12:10591. doi: 10.1038/s41598-022-14961-w (PMC9217969; doi:10.1038/s41598-022-14961-w)
Supplement: Supplementary file 1 — Supplementary Information. [file 41598_2022_14961_MOESM1_ESM.pdf]

Efficacy and safety of ketone ester infusion to prevent muscle weakness in a mouse model of sepsis-induced critical illness

SUPPLEMENTAL FILE

Authors: Ruben Weckx[1], Chloë Goossens[1], Sarah Derde[1], Lies Pauwels[1], Sarah Vander Perre[1], Greet Van den Berghe[1*], Lies Langouche[1*]

Supplemental methods

Supplementary table 1. Overview of gene expression kits

Supplementary figure 1. Cumulative survival of the study

Supplementary figure 2. Impact of increasing doses of pure and racemic enantiomers of 3HHB on hepatic triglyceride content

Supplementary figure 3. Impact of continuous infusion of D,L-3HHB on muscle mass

Supplemental methods

Animal study: In brief, we used 24-week-old C57BL/6J mice (Janvier SAS, Chassal, France) for our validated, centrally catheterized, fluid-resuscitated 5-day model of sustained abdominal sepsis (cecal ligation and puncture) (1). Only male mice were used to avoid hormonal variation due to the estrus cycle in females. Healthy control mice were full-fed receiving standard chow (ssniff R/M-H, ssniff Spezialdiäten Gmbh, Soest, Germany). Mice were randomly allocated to "sepsis" or "healthy control" groups and within the sepsis groups of experiment 1 and 3 randomly allocated to treatment with placebo or ketone ester. Caretakers and data collectors were blinded for group allocation. After surgery, animals were housed in individual house-made transparent swivel cages and placed in a temperature-controlled (27°C) animal cabinet with 12h light and dark cycles. Intravenous fluid resuscitation was started with Plasmalyte A Viaflo (Baxter, Lessines, Belgium) and 6% hydroxyethyl starch in a 4/1 proportion at 0.3 ml/h for the first 20 hours. Six hours post-operatively, mice received subcutaneous antibiotics and analgesia (0.5 mg imipenem/cilastatin, Aurobindo Pharma, Saronno, VA, Italy; and 4.5 µg buprenorphine, Vetergesic, Patheon UK Ltd, Covingham, United Kingdom). Hereafter, antibiotics/analgesia was given every 12 hours until sacrifice (0.5 mg imipenem/cilastatin + 9 µg buprenorphine).

Experiment 1 – Effect of increasing bolus doses of ketone esters on muscle weakness, morbidity and mortality. From day 1 onwards, septic mice received standard mixed parenteral nutrition (PN; Olimel N7E, Baxter) at 5.8 kcal/day supplemented with twice-daily subcutaneous bolus injections of isovolumetric amounts of either D-glucose (6.25 mg/g/day) or ketone ester at the indicated dose for experiment 1.

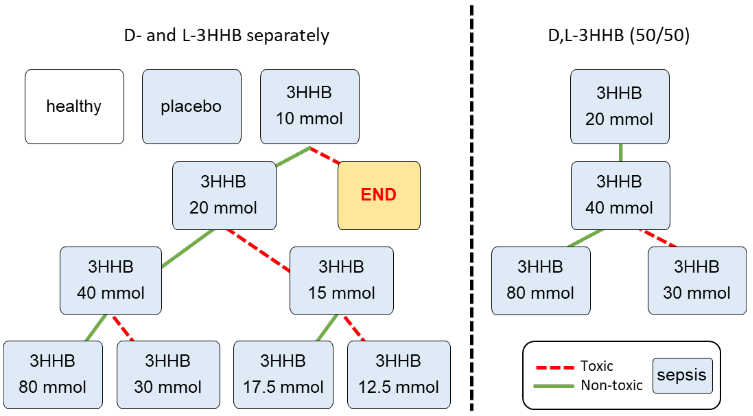

Experimental design experiment 1. Doses are expressed per kg/day.

Experiment 2 – Pharmacokinetic study. From day 1 onwards, septic mice received standard mixed PN (Olimel N7E, Baxter) at 5.8 kcal/day. After subcutaneous bolus injections of D-3HHB at 40 mmol/kg/day and 80 mmol/kg/day at day 2, blood samples were collected at 30, 45, 60 and 180 minutes. Day 2 was chosen to assure illness progression in our mice.

Experiment 3 – effect of continuous administration of ketone esters on muscle weakness, morbidity and mortality. From day 1 onwards, septic mice received standard mixed PN (Olimel N7E, Baxter) at 5.8 kcal/day with continuous 3HHB esters supplementation (40 mmol and 80 mmol/kg/day) or with D-glucose (6.25 mg/kg/day) at a rate of 0.033ml/hour.

A priori defined exclusion criteria of all animal experiments were physical abnormalities present before surgery, death during surgery, pre-randomization death or catheter-malfunction during experiment. Pain/discomfort was assessed twice daily based on the Mouse Grimace Score (2) and scored as: 0 (no discomfort), 1 (moderate discomfort), 2 (severe discomfort). Cumulative illness scores were calculated to assess the severity of illness. Non-surviving mice received the maximal cumulative score +1. Mice were anesthetized and euthanized by cardiac puncture after 2 or 5 days of sepsis, depending on the experiment.

Ex vivo measurement of muscle force: Five day survivors were anaesthetized (intraperitoneal injection of 100 mg/kg ketamine, Eurovet Animal Health BV, Bladel, The Netherlands, and 13 mg/kg xylazine, V.M.D. nv/sa, Arendonk, Belgium) and euthanized via cardiac puncture and decapitation. Immediately thereafter, the extensor digitorum longus (EDL) muscle was isolated and suspended in a temperature controlled and continuously perfused organ bath (30°C, 95% O_2 - 5% CO_2) filled with HEPES-buffered Krebs-ringer solution (0.57 mM $MgSO_4$, 10 mM glucose, 4.5 mM KCl, 120 mM NaCl, 0.7 mM Na_2HPO_4 dibasic, 0.9 mM $Na_2H_2PO_4$ monobasic, 5 mM $MgCl_2$, 1.2 mM KH_2PO_4, 2 mM $CaCl_2$, 10 mM HEPES, pH 7.3). The EDL was mounted vertically between a fixed clamp and lever-arm (300C-LR Dual-Mode muscle lever, Aurora Scientific, Ontario, Canada). Two platinum electrodes, delivering controlled current pulses of 1A (Aurora Scientific), stimulated the muscle. The resting muscle length (L_0) was determined by the highest produced twitch force for each muscle separately. Subsequently, maximal isometric tetanic force was measured by averaging three consecutive tetanic stimuli (180 Hz, 200 ms duration, 0.2 ms pulse width; 2 min rest intervals). The specific maximal isometric tetanic force was determined by dividing the maximal isometric tetanic force by the muscle cross-sectional area (CSA). CSA was calculated by dividing the muscle mass by the product of the density of mammalian skeletal muscle (1.06 mg/mm^3) and the optimal fiber length (L_f=0.44 x L_0).

Tissue analyses

To determine markers of 3HHB metabolism, inflammation and tissue damage in 5-day survivors, RNA was extracted from liver and muscle samples using the RNeasy mini RNA isolation kit (Qiagen, Hilden, Germany) . Samples were homogenized in Qiazol (Qiagen) at 6.5000 rpm for 45 sec with ceramic beads in a Precellys 24 machine (Bertin Technologies, Villeurbanne, France). RNA extraction was performed according to the manufacturer's protocol. RNA concentrations were quantified with a Nanodrop spectrophotometer (ND-1000, Nanodrop Technologies, Wilmington, DE, USA) and reverse-transcribed with Superscript III Reverse Transcriptase (Invitrogen, Merelbeke, Belgium) and random primers

(Invitrogen). Real-time quantification of cDNA was performed with StepOne Plus (Applied Biosystems, Carlsbad, CA, USA) using commercially available TaqMan assays (Applied Biosystems) for all gene expression analyses (Supplementary table 1). Data are shown normalized to 18S ribosomal RNA (*Rn18s*) and were expressed as a fold change of the mean of control mice. To assess histological damage in liver, for 5-day survivors, hematoxylin and eosin stained formalin fixed paraffin liver sections were semi-quantitatively assessed for changes in lipid accumulation, infiltration of inflammatory cells, sinusoidal dilatation and loss of structure (3). Tissue sections were scored as 0, 1 or 2 for minimal (<10%), mild (10-20%) or severe (>20%) abundance of aforementioned parameters except for loss of structure which was scored 0 (not present) or 1 (present). These scores were combined into an overall damage score using the following formula: Hepatic damage score = lipid accumulation (0-2) + inflammation (0-2) + dilatation (0-2) + 2x loss of structure (0-1). The damage score ranges from 0-8 and is rescaled to a 0-2 score scale where 0-1 = 0 (minimal); 2-4 = 1 (mild); 5-8 = 2 (severe). Two independent observers performed scoring and reached consensus in case of scoring discrepancies.

Supplementary table 1. Overview of gene expression kits

Gene symbol	Alternative name	Product name (Applied Biosystems)
Rn18s	18S ribosomal RNA	Mm03928990_g1
Trim63	Tripartite motif-containing 63	Mm01185221_m1
Fbxo32	F-box protein 32	Mm00499523_m1
Adh1	Alcohol dehydrogenase 1 (class I)	Mm00507711_m1
Aldh3b2	Aldehyde dehydrogenase 3 family, member B2	Mm01236535_g1
Aldh1a7	Aldehyde dehydrogenase family 1, subfamily A7	Mm00496380_m1

Supplemental figures

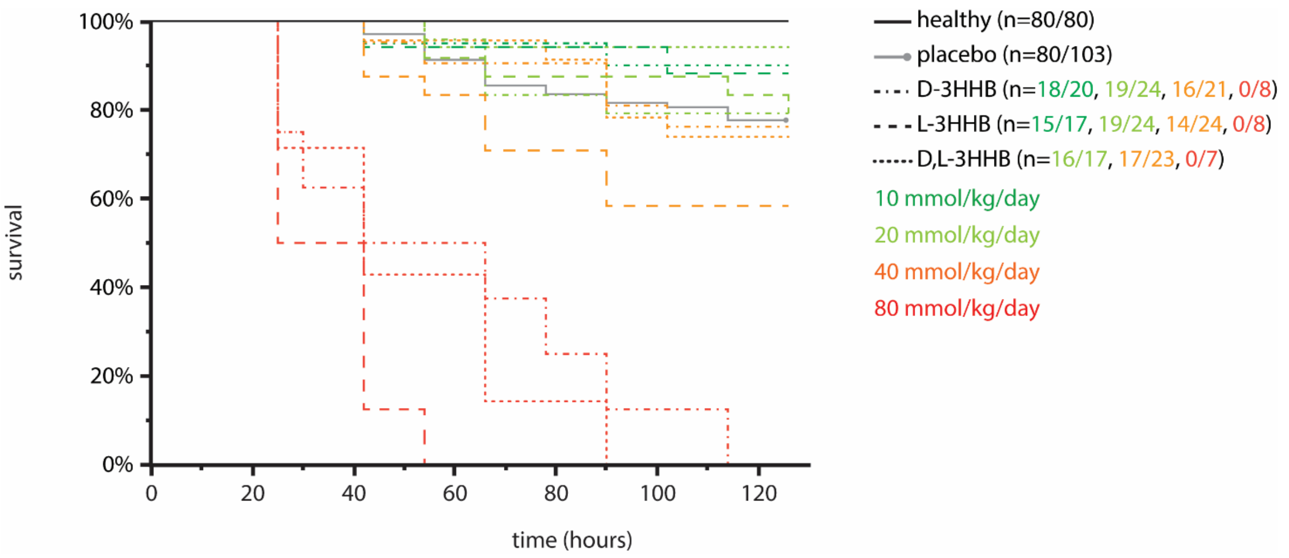

Supplementary figure 1. Cumulative survival of the study. Number of animals is reported as n=survivors/total.

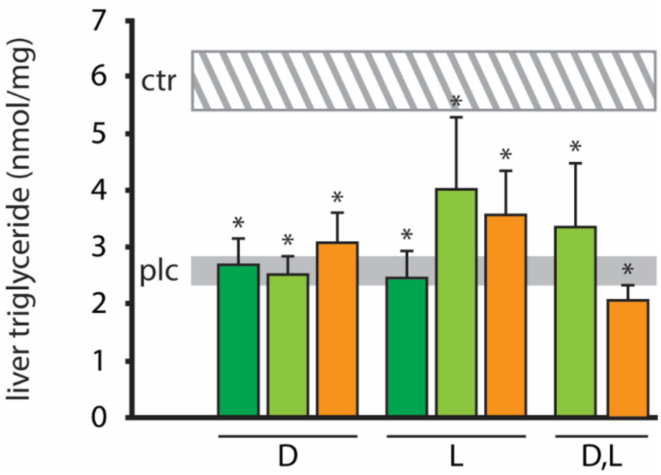

Supplementary figure 2. Impact of increasing bolus doses of pure and mixed racemic enantiomers of 3HHB on hepatic triglyceride content. Data are shown as mean ± standard error of the mean. Horizontal bars: white-grey shaded, healthy control (n=80) grey, placebo (n=74); vertical bars: D-3HHB: 10 mmol/kg/day (n=18); 20 mmol/kg/day (n=19); 40 mmol/kg/day (n=16); L-3HHB: 10 mmol/kg/day (n=15); 20 mmol/kg/day (n=19); 40 mmol/kg/day (n=14); D,L-3HHB: 20 mmol/kg/day (n=16); 40 mmol/kg/day (n=17). ctr: healthy control, plc: placebo, 3HHB: 3-hydroxybutyl-3-hydroxybutanoate ester. * p≤0.05 vs healthy control.

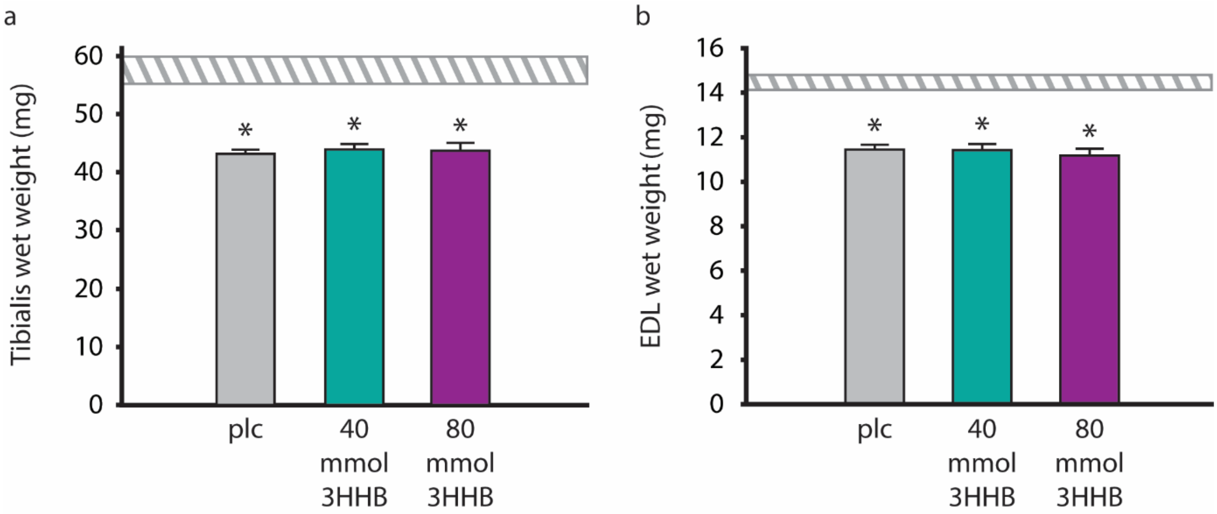

Supplementary figure 3. Impact of continuous infusion of D,L-3HHB on muscle mass. Data are shown as mean ± standard error of the mean. White-grey shaded horizontal bar, healthy control (n=15); vertical bars: plc (n=15); D,L-3HHB: 40 mmol/kg/day (n=17); 80 mmol/kg/day (n=14). plc: placebo, 3HHB: 3-hydroxybutyl-3-hydroxybutanoate ester. * p≤0.05 vs healthy control

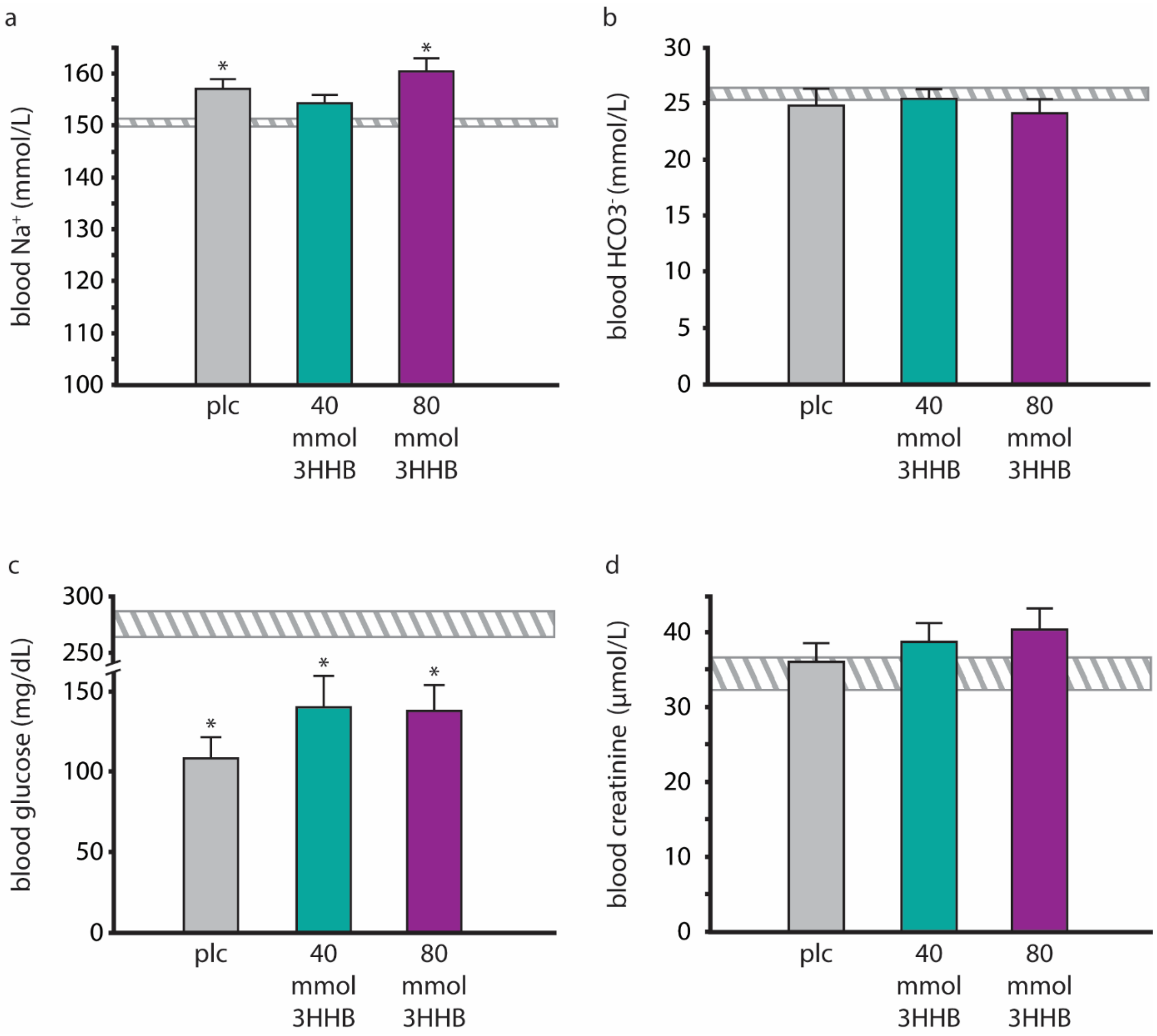

Supplementary figure 4. Impact of continuous infusion of D,L-3HHB on parameters of metabolism and organ function. a, blood Na^+ **b**, blood $HCO3^-$ **c**, blood glucose and **d**, blood creatinine measured at sacrifice. Data are shown as mean ± standard error of the mean. White-grey shaded horizontal bar, healthy control (n=15); vertical bars: plc (n=15); D,L-3HHB: 40 mmol/kg/day (n=17); 80 mmol/kg/day (n=14). plc: placebo, 3HHB: 3-hydroxybutyl-3-hydroxybutanoate ester. * p≤0.05 vs healthy control.

References

1. Derde S, Thiessen SE, Goossens C, Dufour T, Van den Berghe G, Langouche L. Use of a central venous line for fluids, drugs and nutrient administration in a mouse model of critical illness. J Vis Exp (123), e55553, doi:103791/55553 (2017).
2. Langford DJ, Bailey AL, Chanda ML, Clarke SE, Drummond TE, Echols S, et al. Coding of facial expressions of pain in the laboratory mouse. Nat Methods. 2010;7(6):447-9.
3. Jenniskens M, Guiza F, Oorts M, Perre SV, Derde S, Dufour T, et al. On the Role of Illness Duration and Nutrient Restriction in Cholestatic Alterations that Occur During Critical Illness. Shock. 2017.
